# The impact of glucosamine on age-related macular degeneration in patients: A nationwide, population-based cohort study

**DOI:** 10.1371/journal.pone.0251925

**Published:** 2021-05-19

**Authors:** Kathy Ming Feng, Wu-Chien Chien, Jiann-Torng Chen, Yi-Hao Chen, Chi-Hsiang Chung, Chien-An Sun, Ching-Long Chen

**Affiliations:** 1 Department of Ophthalmology, Tri-Service General Hospital, National Defense Medical Center, Taipei, Taiwan; 2 Department of Medical Research, Tri-Service General Hospital, National Defense Medical Center, Taipei, Taiwan; 3 School of Public Health, National Defense Medical Center, Taipei, Taiwan; 4 Taiwanese Injury Prevention and Safety Promotion Association, Taipei, Taiwan; 5 Graduate Institute of Life Sciences, National Defense Medical Center, Taipei, Taiwan; 6 Department of Public Health, College of Medicine, Fu-Jen Catholic University, New Taipei City, Taiwan; 7 Big Data Research Center, College of Medicine, Fu-Jen Catholic University, New Taipei City, Taiwan; National Yang-Ming University Hospital, TAIWAN

## Abstract

**Purpose:**

To analyze the association between glucosamine (GlcN) use and the risk of age-related macular degeneration (AMD) using claims data from the National Health Insurance Research Database (NHIRD).

**Methods:**

A retrospective, population-based study was conducted with NHIRD data from a 14-year period (2000–2013). Chi-squared and Student’s t-tests were used to evaluate differences between the study and comparison cohorts for categorical and continuous variables, respectively. Risk factors for disease development were examined by the adjusted hazard ratio (aHR) with 95% confidence interval. Kaplan-Meier analysis was performed to compare the cumulative risk of AMD between the two cohorts.

**Results:**

In total, 1,344 patients with GlcN treatment were enrolled in the study cohort and 5,376 patients without GlcN use were enrolled in the comparison cohort. The incidence rate of AMD was lower with GlcN use (3.65%) than without GlcN use (5.26%) (P = 0.014). GlcN use was associated with a lower risk of developing AMD among patients with hyperlipidemia, coronary artery disease, chronic obstructive pulmonary disease, stroke, other neurological disorders, or degenerative arthritis. Although the incidence of wet type AMD did not significantly differ (P = 0.91), the incidence of dry type AMD was lower in patients with GlcN use (2.9%) than those without GlcN use (4.84%) (P = 0.003). Kaplan-Meier analysis similarly revealed a lower rate of dry type AMD in patients with GlcN use compared to those without GlcN use (log-rank P = 0.004).

**Conclusions:**

GlcN treatment can decrease the risk of developing dry type AMD. Further prospective controlled studies are needed to determine the effectiveness of GlcN treatment in patients with AMD and the associated mechanism.

## Introduction

Age-related macular degeneration (AMD) is the leading cause of irreversible visual impairment in the developed world, especially among those older than 50 years [1]. In terms of the worldwide prevalence, the predicted number of people with AMD will increase from 196 million in 2020 to 288 million in 2040 [2]. AMD places a heavy burden on patients, caregivers, and physicians [3]. In Taiwan, AMD is a common eye disease among the elderly, and age is the most significant factor associated with AMD [4].

Clinically, AMD is classified into two types: dry (non-neovascular) and wet (neovascular). The dry type is predominant, accounting for 85–90% of all patients with AMD. The early stage of dry AMD causes mild vision loss, and drusen may appear in the macula under fundus examination [5]. Subsequently, it may progress to geographic atrophy (GA), causing irreversible visual impairment [6]. The wet type affects approximately 10–15% of all patients with AMD, but accounts for 90% of cases of vision loss among patients with AMD [7].

Glucosamine (GlcN), a naturally occurring amino monosaccharide, is the most commonly taken dietary supplement; its long-term administration is considered safe in humans [8]. GlcN has been widely used as an alternative regimen for rheumatoid arthritis and osteoarthritis [9, 10]. Our previous *in vitro* and *in vivo* studies have shown that GlcN plays many important roles in anti-inflammatory effects [11–13]. In addition, GlcN modulates oxidative stress-induced senescence of retinal pigment epithelium (RPE) cells [14]. GlcN also attenuates native photoreceptor outer segment (POS)-derived lipofuscin-like autofluorescence (LLAF) in RPE cells *in vitro* [15]. In Taiwan, GlcN is one of the prescription drugs for the alleviation of degenerative arthritis pain insured by the health administration. According to our literature review, no previous studies have investigated the association between GlcN use and AMD. Thus, the purpose of this study was to evaluate the impact of GlcN use on the risk of developing AMD in Taiwan by using claims data from the National Health Insurance Research Database (NHIRD).

## Materials and methods

### Data sources

We utilized outpatient data from the Longitudinal Health Insurance Database (LHID) (2000–2013) of the NHIRD to investigate the association between GlcN treatment and the subsequent development of AMD in Taiwan over a 14-year period. The claims data includes medical information of all those insured, with a coverage rate of more than 99% of the 23 million people in Taiwan. In this study, the medical diagnoses were determined according to the International Classification of Diseases, Ninth Revision, Clinical Modification (ICD-9-CM).

### Study design and sampled participants

We conducted a retrospective matched-cohort study. The study cohort comprised patients who first received GlcN therapy between January 2000 and December 2013. The index date was defined as the date that the patient first received GlcN therapy. The exclusion criteria were as follows: received GlcN treatment before 2000, diagnosed with AMD before receiving GlcN treatment, diagnosed with central serous chorioretinopathy (ICD-9-CM code 362.41) and pathologic myopia (ICD-9-CM code 360.21), received Bilimycin, aged <50 years, and unknown gender. To construct a comparison cohort, 4-fold propensity score matching was applied, randomly selecting patients without GlcN usage who matched those who received GlcN according to gender, age, and index year (under the same exclusion criteria). The tracking endpoint was defined as the date of AMD onset or the end of the study period. AMD was identified by the following ICD-9-CM codes: 362.50, 362.51, 362.52, and 362.57. AMD types were identified as follows: wet type AMD: ICD-9-CM codes 362.52; dry type AMD: ICD-9-CM codes 362.50, 362.51, and 362.57.

### Covariates

The evaluated covariates included gender, age, diabetes (ICD-9-CM codes 250), hypertension (ICD-9-CM codes 401–405), hyperlipidemia (ICD-9-CM codes 272), coronary artery disease (CAD; ICD-9-CM codes 410–414), asthma (ICD-9-CM codes 493), chronic obstructive pulmonary disease (COPD; ICD-9-CM codes 491, 492, and 496), stroke (ICD-9-CM codes 430–438), tobacco dependency (ICD-9-CM code 305.1), heart failure (HF; ICD-9-CM codes 428), dementia (ICD-9-CM codes 290), other neurological disorders (ICD-9-CM codes 344 and 342), degenerative arthritis (ICD-9-CM codes 715) and the Charlson Comorbidity Index (CCI). The number of ophthalmic outpatient visits was adjusted to minimize the surveillance bias.

### Statistical analysis

All analyses were performed using SPSS software version 22 (SPSS Inc., Chicago, Illinois, USA). Chi-squared and t-tests were used to evaluate differences between the study and comparison cohort for categorical and continuous variables, respectively. Multivariable Cox proportional hazards regression analysis was used to determine the risk of AMD, and the results are presented as the adjusted hazard ratio (aHR) with 95% confidence interval (CI). The difference in the cumulative risk of AMD between the study and comparison cohorts was investigated using the Kaplan-Meier method with the log-rank test. A two-tailed p value <0.05 was considered statistically significant.

### Ethics

This study was conducted in accordance with the Code of Ethics of the World Medical Association (Declaration of Helsinki). Patient consent was not required to access the data in the NHIRD. The Institutional Review Board of the Tri-Service General Hospital approved this study (TSGHIRB: B-109-42) and waived the need for individual written informed consent.

## Results

**Fig 1:**
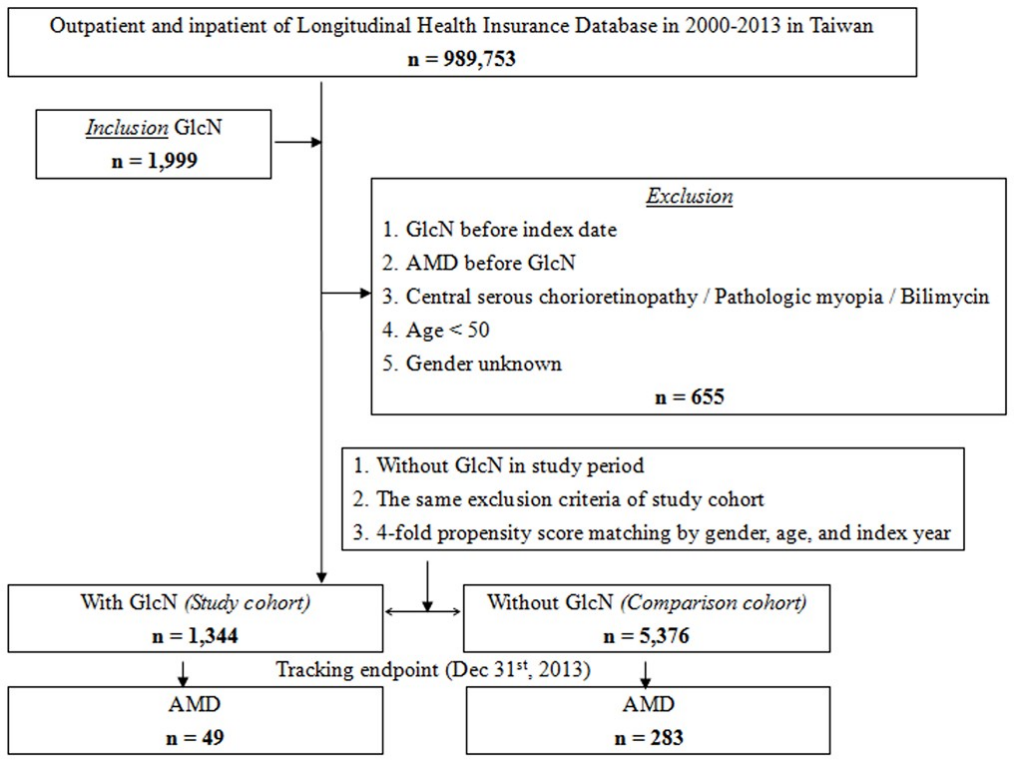
Flowchart of patient selection from the National Health Insurance Research Database in Taiwan.

Among a total of 989,753 patients in the LHID during the study period, 1999 patients received GlcN treatment. Among these, 655 patients were excluded based on the exclusion criteria; thus, 1344 patients were enrolled in the study cohort. After 1:4 matching, 5376 patients without GlcN treatment were enrolled in the comparison cohort. The mean age at baseline was 72.25±16.89 years and 72.31±16.97 years for the study and comparison cohorts, respectively. There were no significant differences in the age at baseline and the gender distribution between the study and comparison cohorts. The study flowchart is depicted in Fig 1.

### Patient characteristics

As shown in Table 1, at the tracking endpoint, the incidence of newly developed AMD was significantly lower with GlcN use (3.65%, 49/1344 patients) than without GlcN use (5.26%, 283/5376 patients) (P = 0.014). Furthermore, the incidence of wet type AMD did not significantly differ between the two cohorts (P = 0.91), whereas the incidence of dry type AMD was significantly lower in patients with GlcN use (2.9%) than those without GlcN use (4.84%) (P = 0.003).

**Table 1:** Patient characteristics at tracking endpoint.

GlcN	Total		With		Without		*P*
Variables	n	%	n	%	n	%	
**Total**	6,720		1,344	20.00	5,376	80.00	
**AMD**							0.014
Without	6,388	95.06	1,295	96.35	5,093	94.74	
With	332	4.94	49	3.65	283	5.26	
**AMD subgroup**							0.011
Without	6,388	95.06	1,295	96.35	5,093	94.74	0.017
Wet type	33	0.49	10	0.74	23	0.43	0.910
Dry type	299	4.45	39	2.90	260	4.84	0.003
**Gender**							0.999
Male	3,125	46.50	625	46.50	2,500	46.50	
Female	3,595	53.50	719	53.50	2,876	53.50	
**Age (yrs)**	75.08 ± 9.34		78.14 ± 7.17		74.31 ± 9.66		<0.001
**Age group (yrs)**							<0.001
50–59	203	3.02	10	0.74	193	3.59	0.155
60–69	1,148	17.08	183	13.62	965	17.95	0.036
70–79	2,490	37.05	572	42.56	1,918	35.68	0.003
≧80	2,879	42.84	579	43.08	2,300	42.78	0.897
**DM**							<0.001
Without	4,569	67.99	705	52.46	3,864	71.88	
With	2,151	32.01	639	47.54	1,512	28.13	
**HTN**							0.961
Without	3,414	50.80	682	50.74	2,732	50.82	
With	3,306	49.20	662	49.26	2,644	49.18	
**Hyperlipidemia**							<0.001
Without	4,600	68.45	675	50.22	3,925	73.01	
With	2,120	31.55	669	49.78	1,451	26.99	
**CAD**							<0.001
Without	4,690	69.79	760	56.55	3,930	73.10	
With	2,030	30.21	584	43.45	1,446	26.90	
**Asthma**							<0.001
Without	5,857	87.16	1,056	78.57	4,801	89.30	
With	863	12.84	288	21.43	575	10.70	
**COPD**							<0.001
Without	4,603	68.50	677	50.37	3,926	73.03	
With	2,117	31.50	667	49.63	1,450	26.97	
**Stroke**							<0.001
Without	5,012	74.58	823	61.24	4,189	77.92	
With	1,708	25.42	521	38.76	1,187	22.08	
**Tobacco dependency**							0.097
Without	6,672	99.29	1,326	98.66	5,346	99.44	
With	48	0.71	18	1.34	30	0.56	
**HF**							<0.001
Without	5,861	87.22	1,059	78.79	4,802	89.32	
With	859	12.78	285	21.21	574	10.68	
**Dementia**							0.003
Without	6,066	90.27	1,141	84.90	4,925	91.61	
With	654	9.73	203	15.10	451	8.39	
**Other neurological disorders**							0.794
Without	6,544	97.38	1,300	96.73	5,244	97.54	
With	176	2.62	44	3.27	132	2.46	
**Degenerative arthritis**							<0.001
Without	5,377	80.01	199	14.81	5,178	96.32	
With	1,343	19.99	1,145	85.19	198	3.68	
**CCI_R**	0.19 ± 0.77		0.13 ± 0.64		0.20 ± 0.80		0.003
**Number of NHI claims for ophthalmic outpatient visits**	7.86 ± 8.02		8.02 ± 8.11		7.85 ± 8.01		0.443

*P*: Chi-square / Fisher exact test on category variables, t-test on continue variables, and proportional test for percentage. GlcN: glucosamine; AMD: age-related macular degeneration; DM: diabetes mellitus; HTN: hypertension; CAD: coronary artery disease; COPD: chronic obstructive pulmonary disease; HF: heart failure; CCI: Charlson comorbidity index.

Additionally, the mean age at the tracking endpoint was higher among patients with GlcN use (78.14±7.17 years) than those without GlcN use (74.31±9.66 years) (P<0.001). The percentage of patients in the age groups of 50–59 years and ≧80 years did not significantly differ between the two cohorts, whereas that for the age groups of 60–69 years and 70–79 years significantly differed between the two cohorts (P = 0.036 and P = 0.003, respectively). In addition, the rates of comorbid DM, hyperlipidemia, CAD, Asthma, COPD, stroke, HF, dementia, and degenerative arthritis, were significantly higher in patients with GlcN use than those without GlcN use. The CCI scores were lower in patients with GlcN use than patients without GlcN use (P = 0.003). The number of NHI claims for ophthalmic outpatient visits was 8.02 ± 8.11 in patients with GlcN use and 7.85 ± 8.01 in those without GlcN use. There was no significant difference between two cohorts (P = 0.443).

### Cumulative risk of AMD by Kaplan-Meier analysis

**Fig 2:**
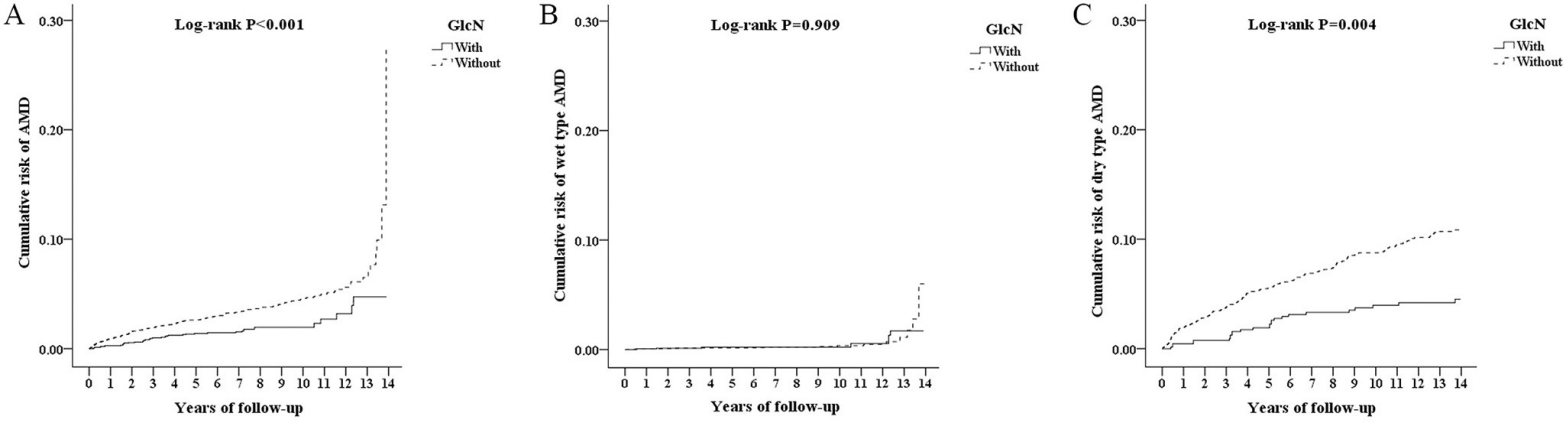
Cumulative risk of AMD in patients stratified by GlcN use, calculated by the Kaplan-Meier method with log-rank test. (A) all AMD; (B) Wet type AMD; (C) Dry type AMD. AMD, Age-related macular degeneration; GlcN, Glucosamine.

As shown in Fig 2A, the cumulative risk of AMD at the end of the 14-year follow-up period was significantly lower in patients with GlcN use than those without GlcN use (log-rank P <0.001). The result of the cumulative risk in AMD subtypes showed that wet type AMD did not significantly differ between the two cohorts (Fig 2B, log-rank P = 0.909), but dry type AMD was significantly lower in patients with GlcN use than those without GlcN use (Fig 2C, log-rank P = 0.004).

### Factors associated with the development of AMD by univariate and multivariate analyses

The results of univariate and multivariate analyses for the risk factors associated with the development of AMD are shown in Table 2. After adjustment for age, gender, comorbidities, and the number of ophthalmic outpatient visits, patients with GlcN use showed a decreased risk for developing AMD compared to that for those without GlcN use (aHR = 0.756; *p* = 0.009).

**Table 2:** Factors associated with the development of AMD as evaluated by Cox regression.

Variables	Crude HR	95% CI	95% CI	*P*	Adjusted HR	95% CI	95% CI	*P*
**GlcN**								
Without	Reference				Reference			
With	0.507	0.374	0.688	<0.001	0.756	0.581	0.919	0.009
**Gender**								
Male	1.709	0.869	1.338	0.491	1.149	0.909	1.464	0.286
Female	Reference				Reference			
**Age group (yrs)**								
50–59	Reference				Reference			
60–69	1.428	1.099	1.856	0.008	1.320	0.953	1.708	0.129
70–79	1.446	1.005	1.831	0.045	1.336	0.984	1.741	0.063
≧80	3.039	2.090	4.418	<0.001	1.889	1.349	2.025	0.007
**DM**								
Without	Reference				Reference			
With	1.553	1.410	1.694	<0.001	1.768	0.591	2.006	0.063
**HTN**								
Without	Reference				Reference			
With	1.646	1.360	1.598	<0.001	1.847	0.633	2.096	0.287
**Hyperlipidemia**								
Without	Reference				Reference			
With	0.530	0.408	0.688	<0.001	0.687	0.530	0.901	0.003
**CAD**								
Without	Reference				Reference			
With	0.356	0.269	0.471	<0.001	0.576	0.427	0.787	<0.001
**Asthma**								
Without	Reference				Reference			
With	0.413	0.275	1.260	0.297	1.122	0.754	1.894	0.529
**COPD**								
Without	Reference				Reference			
With	1.301	1.244	1.402	<0.001	1.403	1.285	1.528	<0.001
**Stroke**								
Without	Reference				Reference			
With	1.306	1.221	1.425	<0.001	1.535	1.383	1.751	<0.001
**Tobacco dependency**								
Without	Reference				Reference			
With	0.688	0.172	2.746	0.588	1.032	0.255	4.224	0.925
**HF**								
Without	Reference				Reference			
With	0.792	0.197	1.488	0.245	1.172	1.003	1.930	0.047
**Dementia**								
Without	Reference				Reference			
With	0.862	0.089	2.314	0.358	1.269	1.135	1.555	<0.001
**Other neurological disorders**								
Without	Reference				Reference			
With	0.288	0.097	0.897	0.034	0.487	0.182	1.451	0.198
**Degenerative arthritis**								
Without	Reference				Reference			
With	1.986	0.876	2.897	0.702	1.865	0.732	2.786	0.711
**CCI_R**	1.323	0.975	2.633	0.314	1.059	0.450	1.778	0.882
**Number of NHI claims for ophthalmic outpatient visits**	1.009	1.001	1.018	0.046	1.004	0.993	1.010	0.055

HR = hazard ratio, CI = confidence interval, Adjusted HR: Adjusted variables listed in the table. GlcN: glucosamine; AMD: age-related macular degeneration; DM: diabetes mellitus; HTN: hypertension; CAD: coronary artery disease; COPD: chronic obstructive pulmonary disease; HF: heart failure; CCI: Charlson comorbidity index.

Additionally patients aged ≧80 years showed an increased risk for developing AMD (aHR = 1.889; *p* = 0.007) compared to those aged 50–59 years. Interestingly, patients with hyperlipidemia or CAD showed a decreased risk of developing AMD, whereas those with COPD, stroke, HF, or dementia showed an increased risk of developing AMD compared to those without these comorbidities. In addition, the risk of developing AMD was affected in Male or patients with comorbidities (HTN, Asthma, tobacco dependency, other neurological disorders, or degenerative arthritis), but there was no statistical significance.

### Stratified analyses comparing the risk of developing AMD between the two cohorts according to background factors

Table 3 provides the results of stratified analyses comparing the risk of developing AMD between patients with and without GlcN use according to each evaluated variable. The risk of developing AMD was lower in patients with GlcN use than those without GlcN use for both gender and for 60–69 and 70–79 age groups; however, the aHR increased with increasing age. Furthermore, the risk of developing AMD was lower in patients with GlcN use than patients without GlcN use among those with comorbidities (hyperlipidemia and CAD), or without comorbidities (DM, HTN, asthma, tobacco dependency, HF, and dementia). Besides, regardless of patients with or without comorbidities (COPD, stroke, other neurological disorders, and degenerative arthritis), patients with GlcN use could decrease the risk of developing AMD compared to those without GlcN use.

**Table 3:** Stratified Cox regression analyses comparing the risk of developing AMD between the two cohorts according to background factors.

GlcN	With			Without			With *vs*. Without *(Reference)*				
Stratified	Events	PYs	Rate (per 10^5^ PYs)	Events	PYs	Rate (per 10^5^ PYs)	Ratio	Adjusted HR	95% CI	95% CI	*P*
**Total**	49	15,375.87	318.68	283	39,259.07	720.85	0.442	0.756	0.581	0.919	0.009
**Gender**											
Male	26	5,631.56	461.68	152	17,298.14	878.71	0.525	0.899	0.690	1.092	0.062
Female	23	9,744.31	236.04	131	21,960.93	596.51	0.396	0.676	0.520	0.822	<0.001
**Age group (years)**											
50–59	0	1,233.08	0.00	39	1,783.28	2,186.98	0.000	0.000	-	-	0.997
60–69	6	2,759.74	217.41	60	6,258.46	958.70	0.227	0.388	0.298	0.471	<0.001
70–79	19	7,164.46	265.20	107	16,620.06	643.80	0.412	0.704	0.541	0.856	0.006
≧80	24	4,218.59	568.91	77	14,597.27	527.50	1.079	1.844	0.940	2.241	0.285
**DM**											
Without	33	7,522.00	438.71	224	22,376.23	1,001.06	0.438	0.749	0.576	0.911	0.002
With	16	7,853.87	203.72	59	16,882.83	349.47	0.583	0.998	0.765	1.211	0.156
**HTN**											
Without	17	5,991.86	283.72	155	9,778.49	1,585.11	0.179	0.306	0.235	0.372	<0.001
With	32	9,384.01	341.01	128	29,480.58	434.18	0.785	1.344	0.936	1.632	0.271
**Hyperlipidemia**											
Without	31	5,817.22	532.90	213	21,911.45	972.09	0.548	0.938	0.720	1.139	0.125
With	18	9,558.65	188.31	70	17,347.62	403.51	0.467	0.798	0.612	0.970	<0.001
**CAD**											
Without	31	6,948.29	446.15	200	21,714.05	921.06	0.484	0.829	0.636	1.007	0.053
With	18	8,427.58	213.58	83	17,545.02	473.07	0.451	0.772	0.593	0.939	0.001
**Asthma**											
Without	41	11,752.59	348.86	263	32,644.55	805.65	0.433	0.740	0.569	0.900	<0.001
With	8	3,623.28	220.79	20	6,614.51	302.37	0.730	1.249	0.959	1.517	0.355
**COPD**											
Without	28	6,409.00	436.89	202	21,381.41	944.75	0.462	0.791	0.607	0.961	<0.001
With	21	8,966.87	234.20	81	17,877.66	453.08	0.517	0.884	0.678	0.995	0.048
**Stroke**											
Without	38	9,373.10	405.42	228	24,946.32	913.96	0.444	0.759	0.583	0.922	<0.001
With	11	6,002.77	183.25	55	14,312.74	384.27	0.477	0.816	0.626	0.991	0.042
**Tobacco dependency**											
Without	49	14,938.77	328.01	281	38,920.91	721.98	0.454	0.777	0.597	0.944	0.001
With	0	437.10	0.00	2	338.16	591.44	0.000	0.000	-	-	0.897
**HF**											
Without	39	11,953.40	326.27	238	30,849.79	771.48	0.423	0.723	0.556	0.879	<0.001
With	10	3,422.47	292.19	45	8,409.28	535.12	0.546	0.934	0.717	1.135	0.496
**Dementia**											
Without	49	13,943.65	351.41	280	34,034.57	822.69	0.427	0.730	0.561	0.888	<0.001
With	0	1,432.23	0.00	3	5,224.50	57.42	0.000	0.000	-	-	0.916
**Other neurological disorders**											
Without	47	13,964.03	336.58	277	37,532.02	738.04	0.456	0.780	0.599	0.948	0.026
With	2	1,411.84	141.66	6	1,727.05	347.41	0.408	0.697	0.536	0.847	<0.001
**Degenerative arthritis**											
Without	13	2,078.37	625.49	279	37,929.67	735.57	0.850	0.724	0.488	0.801	<0.001
With	36	13,297.50	270.73	4	1,329.40	300.89	0.900	0.867	0.596	0.948	0.011

PYs: Person-years; Adjusted HR: Adjusted Hazard ratio: Adjusted for the variables listed in Table 2; CI: confidence interval; GlcN: glucosamine; AMD: age-related macular degeneration; DM: diabetes mellitus; HTN: hypertension; CAD: coronary artery disease; COPD: chronic obstructive pulmonary disease; HF: heart failure.

### Stratified analyses according to AMD type

The risk of developing wet type AMD did not significantly differ between two cohorts (aHR = 0.987; P = 0.186), whereas the risk of developing dry type AMD was 0.663-fold less in patients with GlcN use than those without GlcN use (P = 0.009) (Table 4).

**Table 4:** Stratified Cox regression analyses according to AMD type.

GlcN	With			Without *(Reference)*			With *vs*. Without *(Reference)*				
AMD subgroup	Events	PYs	Rate (per 10^5^ PYs)	Events	PYs	Rate (per 10^5^ PYs)	Ratio	Adjusted HR	95% CI	95% CI	*P*
All type	49	15,375.87	318.68	283	39,259.07	720.85	0.442	0.756	0.581	0.919	0.009
Wet type	10	15,375.87	65.04	23	39,259.07	58.59	1.110	0.987	0.746	1.485	0.186
Dry type	39	15,375.87	253.64	260	39,259.07	662.27	0.383	0.663	0.482	0.810	0.009

AMD: age-related macular degeneration; PYs: Person-years; Adjusted HR: Adjusted Hazard ratio: Adjusted for the variables listed in Table 2; CI: confidence interval.

### Association between the duration of GlcN use and the development of AMD

As shown in Table 5, after adjustment for covariates, the duration of GlcN use impacted the development of AMD (all type), but only patient with GlcN use≧3 years has statistical significance (aHR = 0.456; p = 0.011). Furthermore, we analyzed this relationship in the wet and dry type AMD. The result shown that there was no significant association between the druation of GlcN use and the development of wet type AMD. Interestingly, the development of dry type AMD was related to the duration of GlcN use, especially those with GlcN use ≧1 year but <3 year (aHR = 0.742; P = 0.043) and GlcN use ≧3 years (aHR = 0.493; P = 0.003) subgroup. Taken together, this result demonstrated that the effect of GlcN on the reduced risk of developing dry type AMD was depended on the duration of GlcN use.

**Table 5:** Association between the duration of GlcN use and the development of AMD.

AMD subgroup	GlcN usage	Events	PYs	Rate (per 10^5^ PYs)	Crude HR	95% CI	95% CI	*P*	Adjusted HR	95% CI	95% CI	*P*
All type	0 (Without)	283	39,259.07	720.85	Reference				Reference			
	<1 year	33	10,218.30	322.95	0.537	0.309	0.984	0.019	0.779	0.433	1.420	0.416
	≧1 year, <3 years	11	3,220.81	341.53	0.508	0.927	0.929	0.027	0.713	0.389	1.345	0.271
	≧3 years	5	1,936.76	258.16	0.302	0.125	0.732	0.009	0.456	0.183	0.896	0.011
Wet type	0 (Without)	23	39,259.07	58.59	Reference				Reference			
	<1 year	9	10,218.30	88.08	1.036	0.356	3.021	0.924	1.657	0.658	7.121	0.432
	≧1 year, <3 years	1	3,220.81	31.05	0.289	0.036	2.099	0.922	0.593	0.077	4.625	0.621
	≧3 years	0	1,936.76	0.00	0.000	-	-	0.960	0.000	-	-	0.989
Dry type	0 (Without)	260	39,259.07	662.27	Reference				Reference			
	<1 year	24	10,218.30	234.87	0.449	0.227	0.882	0.006	0.606	0.303	1.212	0.142
	≧1 year, <3 years	10	3,220.81	310.48	0.543	0.289	0.954	0.036	0.742	0.389	0.986	0.043
	≧3 years	5	1,936.76	258.16	0.360	0.149	0.702	0.001	0.493	0.199	0.775	0.003

AMD: age-related macular degeneration; PYs: Person-years; Adjusted HR: Adjusted Hazard ratio: Adjusted for the variables listed in Table 2; CI: confidence interval.

## Discussion

The current study revealed that, after adjusting for covariates, patients with GlcN treatment had a lower risk of developing AMD compared to that in matched patients without GlcN treatment. Furthermore, stratified analyses according to AMD subtype showed that patients who used GlcN had a decreased risk of developing dry type AMD, but not wet type AMD, compared to those without GlcN use. Similarly, the Kaplan-Meier analysis revealed that patients with GlcN treatment had a significantly lower risk of dry type AMD than those without GlcN treatment. Interestingly, GlcN use was associated with a decreased risk of developing AMD in both gender, 60–69 and 70–79 age groups, and patients with comorbid hyperlipidemia, CAD, COPD, stroke, other neurological disorders, or degenerative arthritis. We also found that durations of GlcN treatment ≧1 year were associated with decreased risk of dry type AMD. This is the first population-based study to investigate and demonstrate a relationship between GlcN use and the risk of developing AMD, especially dry type AMD.

AMD is known for photoreceptor death and RPE degeneration, with chronic inflammation, whereas GlcN has been shown to decrease inflammation in cellular and animal studies [11–13]. One pathological hallmark of AMD is the degeneration of RPE cells due to an excessive accumulation of lipofuscin, which forms reactive oxygen species. *In vitro* studies have shown that GlcN can induce autophagy through the AMP-activated protein kinase-mammalian target of rapamycin pathway, thereby reducing the increase in LLAF in native POS-treated ARPE-19 cells [15]. Autophagy is a self-destructive process, in which autophagosomes containing unused or damaged intracellular components are delivered to the lysosome for degradation. The timing of autophagy is important. In the late stages of AMD, autophagy may exacerbate the disease if the RPE is damaged past a critical point. A similar concept was performed in an animal model of Alzheimer’s disease (AD) [16].

AMD is one of progressive chronic disease. It progresses slowly from early stage to intermediate stage and ultimately late stage, either neovascular AMD (wet AMD) or geographic atrophy (GA, late stage of dry AMD). The progression of AMD is related to the elevated oxidative stress and lipid peroxidation in RPEcells [17]. Our previous study has reported that GlcN reduced the native POS- induced LLAF in RPE cells, but did not influence lipid peroxidation (Malondialdehyde or 4-hydroxynonenal) -modified POS induced LLAF [15]. In present study, we also found that GlcN use only decreased the risk of developing of dry type AMD, not wet type AMD. This result may mean that GlcN treatment plays a role to slowdown the progression from aging retina to early stages of dry AMD, but not early to late stages of AMD (GA or wet AMD). However, further prospective studies are necessary to confirm this association between GlcN treatment and the subtypes of AMD.

AMD is a multifactorial disease, and includes age, smoking, genetic variants and environmental factors [18–20]. By far, age is the strongest risk factor for AMD [18, 19]. The prevalence of AMD has been reported as 0.2% for those aged 55–64 years and 13.1% for those aged greater than 85 years [18]. Previously, one regional study of Taiwan has reported that the risk of AMD was significantly higher in older populations, with age over 65 years, and the prevalence increased from 5% in those aged 65–69 years to 24.4% in those aged over 80 years [4]. Consistent with previous studies [4, 18, 19], our result also demonstrated that age is a significant risk factor for the development of AMD. In addition, we also found that patients with GlcN use were significantly older than those without GlcN use at the tracking endpoint. To explain this result, we further analyzed the tracking period (between the index date and the tracking endpoint) of the study and comparison cohort. As shown in S1 Table, we found that patients with GlcN use had a longer period to develop AMD (mean±SD = 4.84 ± 3.96 years) than patients without GlcN use (mean±SD = 3.28 ± 3.36). This may explain why patients in comparison cohort (who supposed were age-matched with patients who were prescribed GlcN) were younger than those treated with GlcN at the tracking endpoint. Furthermore, in age groups of 60–69 and 70–79 years, patients with GlcN treatment have a lower risk of AMD than those without GlcN treatment.

In addition, previous studies have shown a higher incidence of AMD in women [4, 18], whereas other studies reported a higher incidence of AMD in men [21, 22]. In this study, our results found that compared to women, men had a slight increase of risk for AMD, but there was no statistical difference (aHR = 1.149, P = 0.286). This result is not consistent to the Shihpai Eye study of Taiwan [4]. One possible explanation was that our study encompassed almost the entire population in Taiwan and the enrolled population was different in studies.

Smoking, one modifiable risk factor, has also been strongly associated with AMD in several population studies [18, 23]. It can increase oxidative stress and vascular endothelial growth factor expression, and activate the immune system, particularly the alternative pathway through the complement system [22, 24]. In our study, we observed an increased risk of AMD in patients with tobacco dependency, but there was no statistical significance (aHR = 1.032, P = 0.925). This might be due to that patients with tobacco dependency are those who are addicted to tobacco to the point of hindering the patient’s social functioning and health, not included regular smokers who may or may not be addicted. Being retrospective in nature, our study could not obtain data regarding the smoking habits of each patient. Furthermore, there was no tobacco dependency patient in our GlcN treatment cohort; hence, it is difficult to interpret the results regarding GlcN use and AMD risk in patients with tobacco dependency.

There are multiple systemic risk factors associated with AMD include obesity, cardiovascular disease, hypertension, asthma, emphysema, and dementia [19, 25, 26]. In present study, we also found that several comorbidities increased the risk of AMD, including COPD, stroke, HF, and dementia compared to patients without these comorbidities. Due to COPD included emphysema and chronic bronchitis, this might explain that COPD was associated with a significantly increased risk of AMD in present study. In addition, patients with GlcN use could decrease the risk of developing AMD in patients with asthma or COPD. One possible explanation was that GlcN possesses anti-inflammatory effect and the pathogenesis of AMD, asthma, and COPD are associated with chronic inflammation [27, 28].

GlcN use was associated with a decreased risk of AMD among patients with CAD, stroke, and hyperlipidemia. A recent prospective study using United Kingdom Biobank data from 466,039 participants similarly found a lower risk of cardiovascular events in GlcN users, including those with CAD (HR: 0.82) and stroke (HR: 0.91) [29]. Another study using United Kingdom Biobank data found that GlcN was also associated with lower mortality in patients with cancer and cardiovascular disease [30]. This could be explained by GlcN’s anti-inflammation effects, with antioxidative stress-induced senescence and induced autophagy as shown in *in vitro* studies [12, 14, 15].

A recent meta-analysis reported a significantly increased risk of AMD in patients with dementia or Alzheimer’s disease (AD), and AMD was associated with cognitive impairment [26]. AD is the most prevalent cause of dementia. The present study results suggest that there is a significantly increased risk of AMD in patients with dementia. Not only AMD and dementia share similar risk factors, but also their pathogeneses show similarities as well. AD is known for the deposition of amyloid β aggregates in the central nervous system and retina, and AMD is known for amyloid β deposits in drusens and the RPE from studies with donor human eyes [26, 31]. A cross-sectional study reported that GlcN is associated with beneficial cognitive function [32] and in *in vivo* studies, GlcN can increase the level of brain-derived neurotrophic factor, which is involved in memory consolidation and cognitive function [33]. With these numerous similarities, one can hypothesize that GlcN may be beneficial for patients with AMD as well; however, further research is needed. It would be interesting to ascertain the risk of AMD in patients with dementia taking a GlcN supplement; the current study did not have patients in this category.

In the present study, patients with GlcN use had a lower prevalence of AMD, with a statistical significance of *p* = 0.005. Due to its immunosuppressive activity, seen *in vitro* and *in vivo* studies, GlcN has been widely used as an alternative supplement in osteoarthritis and rheumatoid arthritis to prevent joint space narrowing and reduce osteoarthritis-related surgeries [34]. Indeed, the prescription of GlcN was covered and regulated by the National Health Insurance (NHI) in Taiwan before 2018 for patients that met the following criteria: age >60 years, Ahlback classification of severity of knee osteoarthritis <stage 3, osteoarthritis symptoms lasting >6 months, and a Lequesne’s severity index for knee osteoarthritis of ≥7 points. A maximum dose of 750 mg/day of GlcN, with 2 courses of a 3-month treatment each year, can be reimbursed by the NHI. Thus, this might been the reason that the rates of degenerative arthritis was significantly higher in patients with GlcN use than those without GlcN use. Moreover, despite patient with or without degenerative arthritis, the result of the stratified analysis demonstrated that patients with GlcN use decreased the risk of developing AMD compared to those without GlcN use. In addition, the duration of GlcN use also affected the risk of AMD. The present study showed that GlcN use for ≥1 year but <3 years, and especially GlcN use for ≥3 years, was associated with a decreased risk of developing dry type AMD. Taken together, these results demonstrate that GlcN use for ≥1 year decreased the risk of dry type AMD, and suggest that the longer glucosamine treatment, the lower the risk of AMD.

To the best of our knowledge, the current study is the first population-based study to evaluate the association of GlcN use with the risk of AMD. Since GlcN was insured by the NHI, we were able to track the duration of GlcN use in our study cohort, an advantage of the current study. We were also able to observe the effect of GlcN in each AMD subtype. Our sample size was large and had long-term follow up, which provided considerable statistical power, and may better reflect the real-world situation than city-based or hospital-based studies.

There are several limitations to our study. First, the NHI stopped funding GlcN prescriptions after October 2018; hence, data could only be obtained before 2018. Secondly, data regarding the dietary habits of each patient could not be obtained; thus, it is unknown whether patients used GlcN supplements by other means. However, it is reasonable to assume that a majority of the patients would not pay the extra cost for GlcN supplements if they could acquire GlcN through the NHI (due to the burden of healthcare costs). Finally, this study was retrospective in nature, and the database lacked imaging examination findings to confirm the diagnosis. Thus, misclassifications are possible. Nevertheless, this study comprised a real-world population study.

In conclusion, this retrospective, population-based cohort study revealed that patients with GlcN use had a lower risk of developing dry type AMD than those without GlcN use. Furthermore, age, COPD, stroke, HF, and dementia were associated with a higher risk of developing AMD; however, GlcN use decreased the risk of AMD in those with older age, hyperlipidemia, CAD, COPD, stroke, other neurologic disorders, or degenerative arthritis. Additionally, the decrease of the developing dry type AMD in patients with GlcN use was depended on the duration of GlcN treatment, especially those with GlcN use ≧1 year. Future well-designed clinical trials are needed to confirm this association between GlcN use and AMD risk.

## Supporting information

S1 Table The tracking period between the index date and the tracking endpoint in the study and comparison cohort. (DOC)
