# Supplementary material for: The impact of glucosamine on age-related macular degeneration in patients: A nationwide, population-based cohort study
Source: PLoS One. 2021 May 19;16(5):e0251925. doi: 10.1371/journal.pone.0251925 (PMC8133402; doi:10.1371/journal.pone.0251925)
Supplement: S1 Table — (DOC) [file pone.0251925.s001.doc]

S1 Table. The tracking period between the index date and the tracking endpoint in the study and comparison cohort

| **GlcN** | **Mean ± SD** |
| --- | --- |
| With | 4.84 ± 3.96 |
| Without | 3.28 ± 3.36 |
| Total | 3.51 ± 3.49 |

GlcN: glucosamine; Study cohort: with GlcN use; Comparison cohort: without GlcN use
